# Supplementary material for: Gestational and Lactation Exposure to Perfluorohexanoic Acid Results in Sex-Specific Changes in the Cerebellum in Mice
Source: Int J Mol Sci. 2025 Aug 19;26(16):8008. doi: 10.3390/ijms26168008 (PMC12387024; doi:10.3390/ijms26168008)
Supplement: Supplementary file 1 [file ijms-26-08008-s001.zip › ijms-3763344-supplementary.pdf]

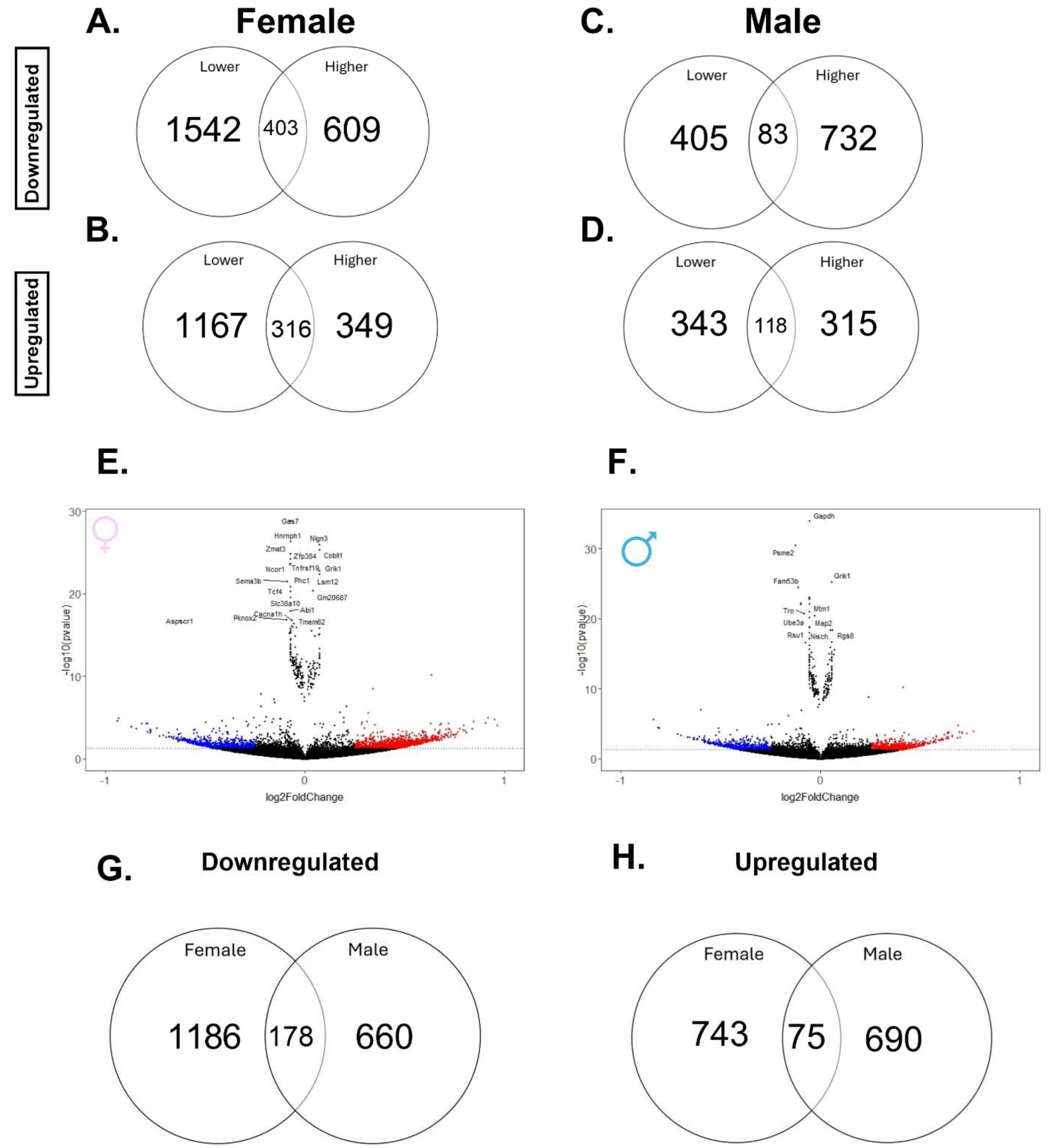

Supplementary Figure S1. PFHxA lower and higher exposure dysregulates different genes. Venn diagrams show common genes downregulated (A,C) and upregulated (B,D) in both the lower and higher exposure groups when each is compared to control in females (A,B) and males (B,C). Volcano plots in (E) females and (F) males show significantly upregulated genes in red and significantly downregulated genes in blue. Venn diagrams show more (G) downregulated and (H) upregulated genes in females as compared to males.

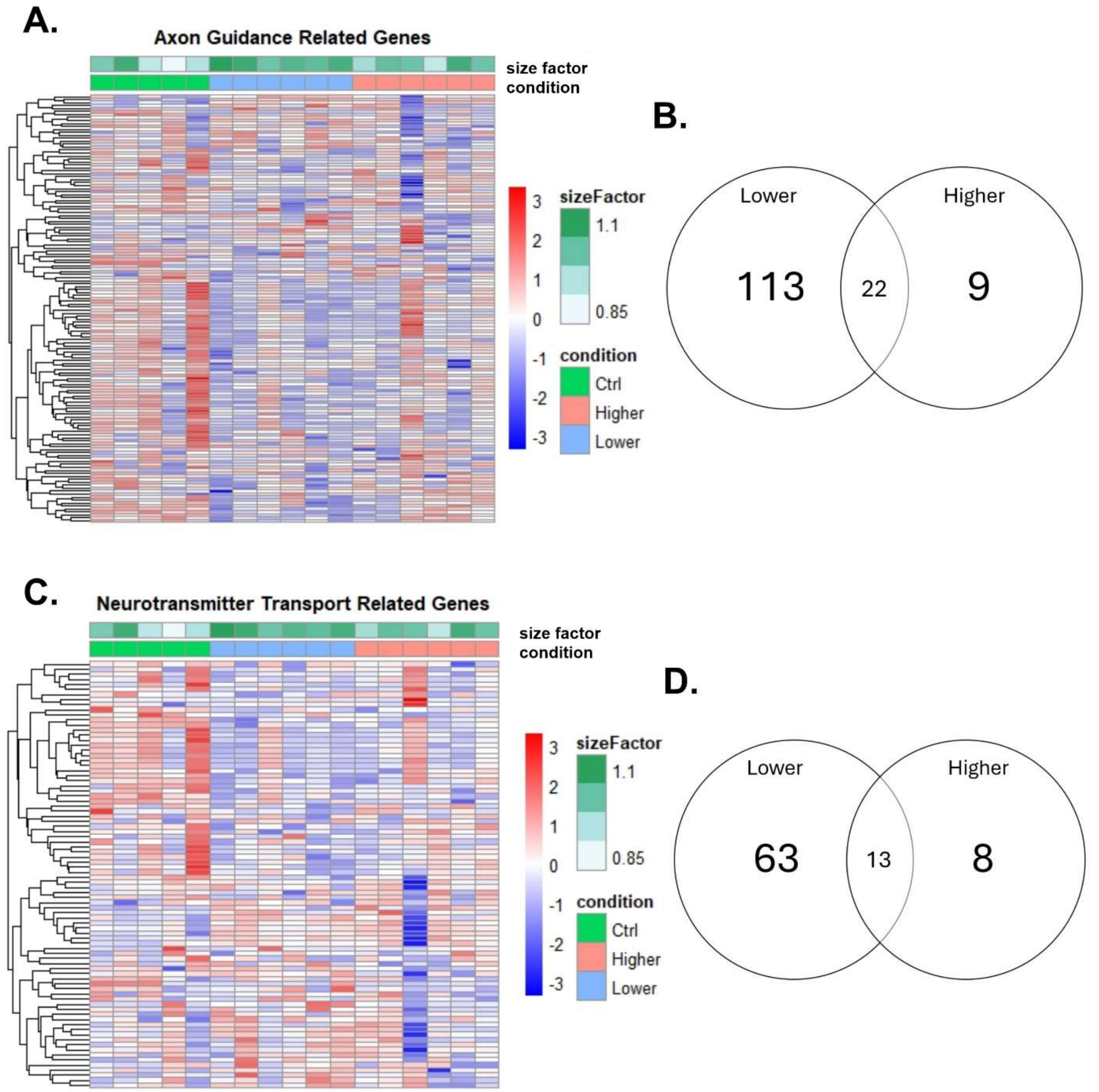

Supplementary Figure S2. (A) Heatmap of significantly dysregulated axon guidance related genes in females in lower or higher exposure groups compared to controls using a pairwise comparison. (B) Number of significantly dysregulated axon guidance genes in each exposure group in females. (C) Heatmap of significantly dysregulated neurotransmitter transport related genes in females in the lower or higher exposure groups compared to controls using a pairwise comparison. (D) Number of significantly dysregulated neurotransmitter transport genes in each

exposure group in females. Size factor is the scaling factor to normalize raw read counts for each sample to account for differences in library size.

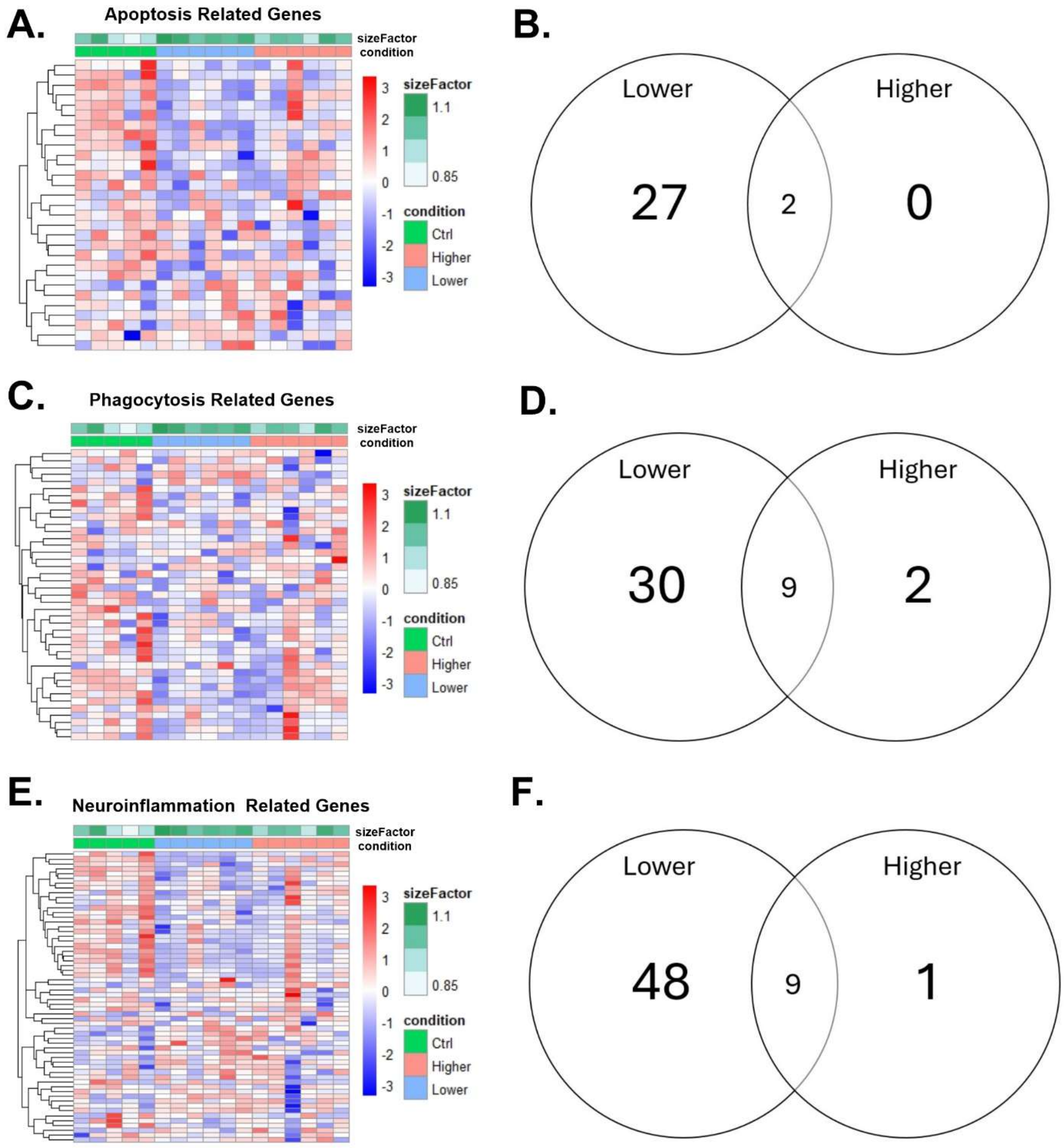

Supplementary Figure S3. PFHxA exposure affects apoptosis, phagocytosis, and neuroinflammation related genes in females. Heatmaps of significantly dysregulated apoptosis (A), phagocytosis (C), and neuroinflammation (E) related genes in lower or higher exposure groups compared to controls using a pairwise comparison. Number of significantly dysregulated

apoptosis (B), phagocytosis (D), and neuroinflammation (F) genes in each exposure group. Size factor is the scaling factor to normalize raw read counts for each sample to account for differences in library size.

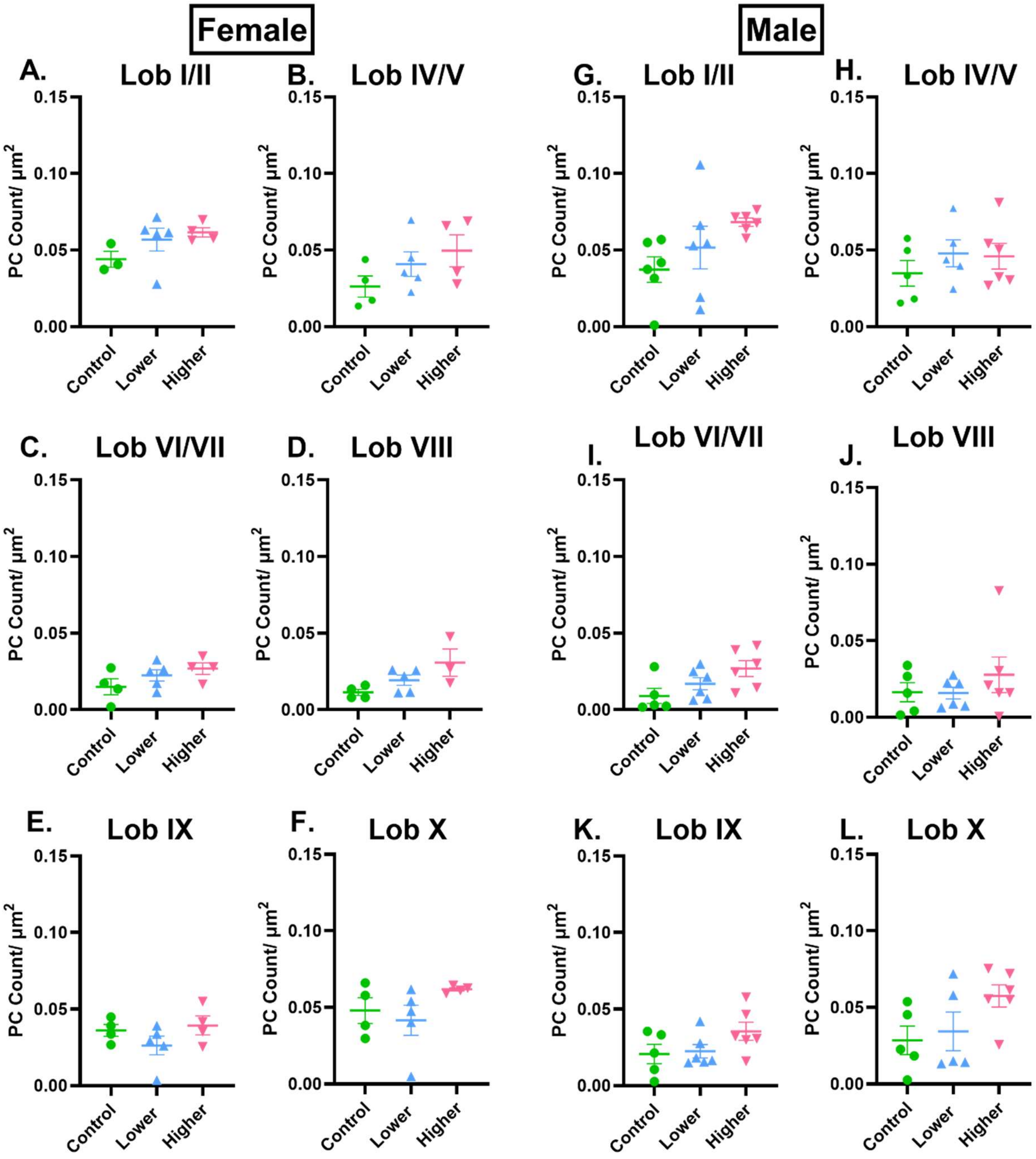

Supplementary Figure S4. PFHxA exposure did not affect Purkinje cell linear frequency in lobules I/II and lobules IV-X. There were no changes in PC linear frequency females in any lobules (A-

F) or in males in any lobules (G-L). Individual points represent individual animals (N=3-6). Data are presented as the mean ±SEM. One-way ANOVA with Tukey post hoc analysis.

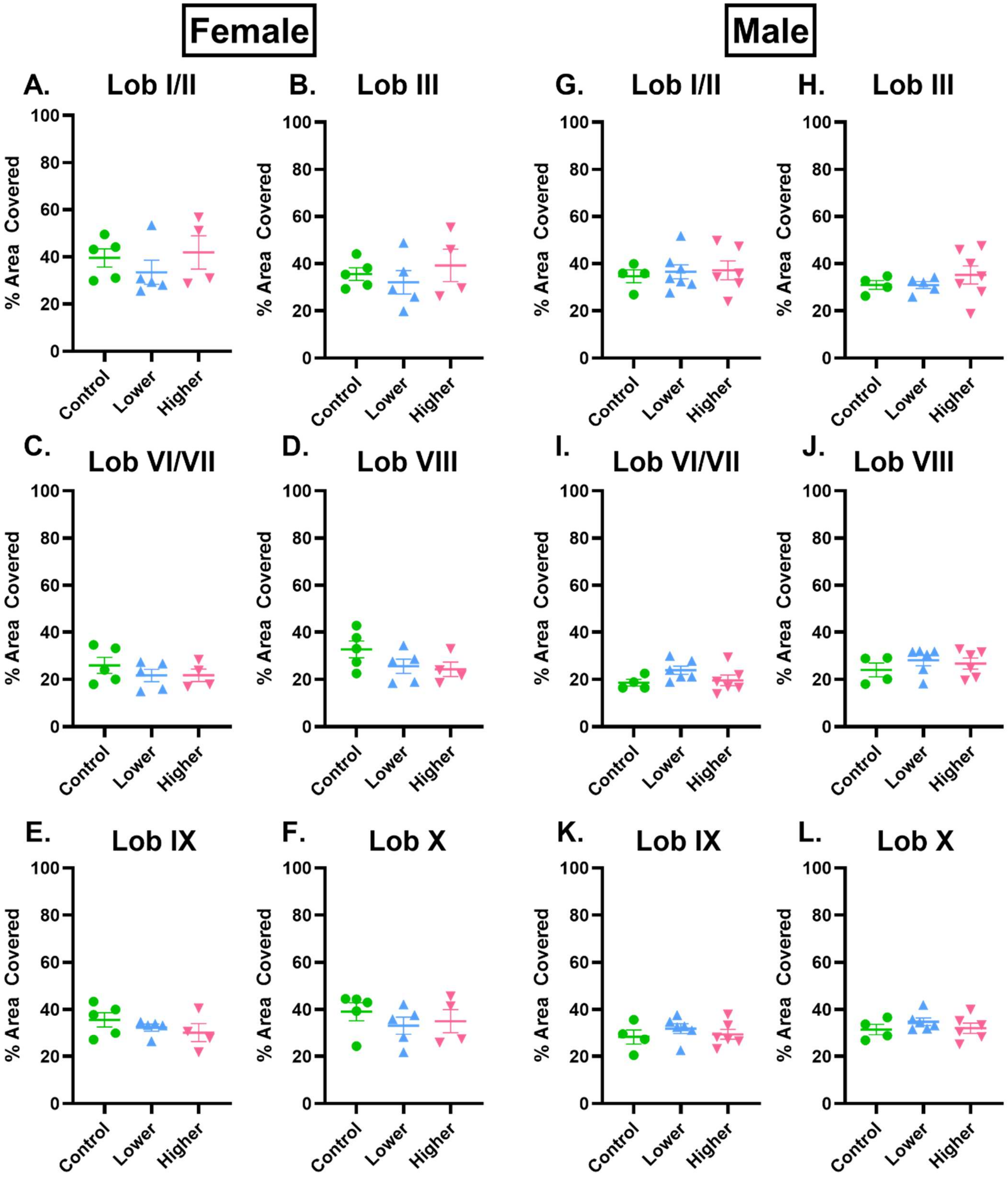

Female
Male
A.
Lob I/II
B.
Lob III
G.
Lob I/II
H.
Lob III
C.
Lob VI/VII
D.
Lob VIII
I.
Lob VI/VII
J.
Lob VIII
E.
Lob IX
F.
Lob X
K.
Lob IX
L.
Lob X
% Area Covered
0
20
40
60
80
100
Control
Lower
Higher

Supplementary Figure S5. PFHxA did not affect MBP percent coverage in any lobule. In females there were no changes in MBP percent coverage in (A) lobule I/II, (B) lobule III, (C) lobule VI/VII, (D) lobule VIII, (E) lobule IX, or (F) lobule X. In males, there were no changes in MBP percent coverage in (G) lobule I/II, (H) lobule III, (I) lobule VI/VII, (J) lobule VIII, (K) lobule IX, or (L) lobule X. Individual points represent individual animals (N=4-6). Data are presented as the mean ±SEM. One-way ANOVA with Tukey *post hoc* analysis.

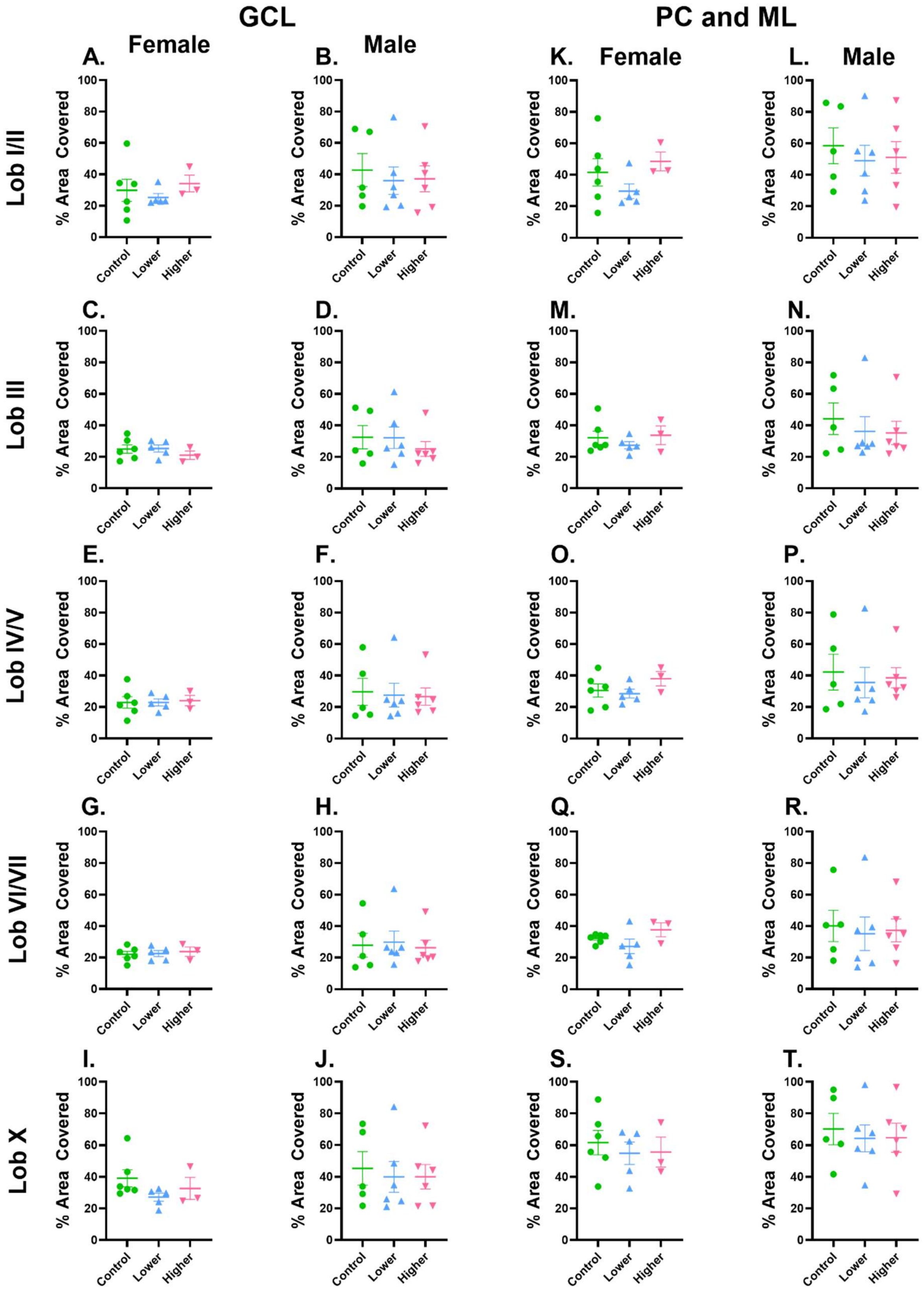

GCL
PC and ML
Female
Male
Lob I/II
Lob III
Lob IV/V
Lob VI/VII
Lob X
A.
B.
C.
D.
E.
F.
G.
H.
I.
J.
K.
L.
M.
N.
O.
P.
Q.
R.
S.
T.
% Area Covered
Control
Lower
Higher

Supplementary Figure S6. PFHxA exposure did not affect GFAP percent coverage in any lobule. There were no changes in percent GFAP coverage in the GCL (A-J) or the PC and ML (K-T) in females or males in (A-B) lobule (lob) I/II, (C-D) lob III, (E-F) lob IV/V, (G-H) lob VI/VII, or (I-J) lob X. Individual points represent individual animals (N=3-6). Data are presented as the mean ±SEM. One-way ANOVA with Tukey *post hoc* analysis.

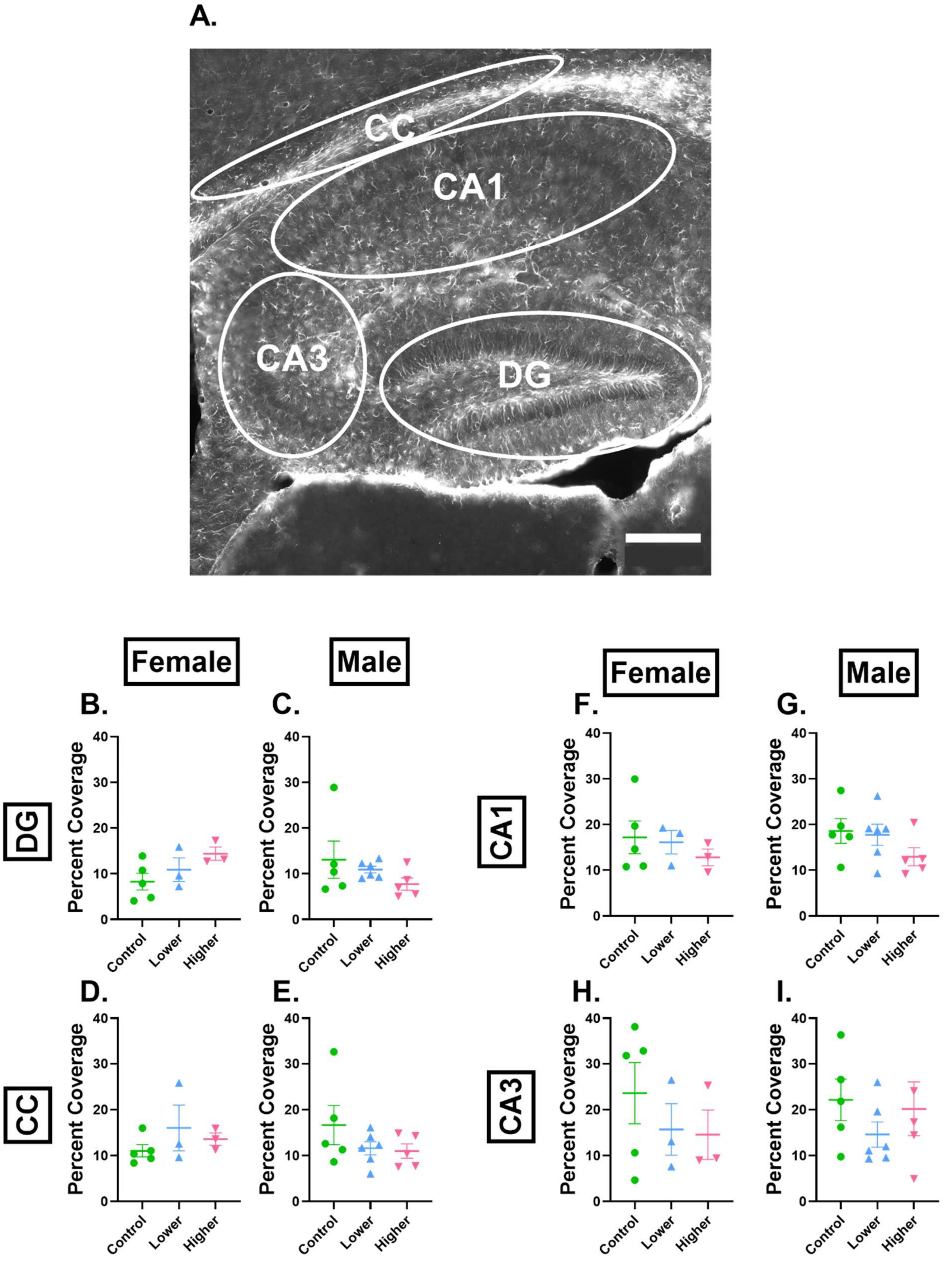
A.
CC
CA1
CA3
DG
Female
Male
Female
Male
B.
C.
F.
G.
DG
CA1
D.
E.
H.
I.
CC
CA3
Percent Coverage
0
10
20
30
40
Control
Lower
Higher

Supplementary Figure S7. PFHxA exposure did not affect GFAP percent coverage in the hippocampus. (A) Representative image of GFAP in the dentate gyrus (DG), corpus callosum (CC), CA1, and CA3. There were no changes in GFAP percent area covered in the DG in (B) females or (C) males, in the CC in (D) females or (E) males, in the CA1 in (F) females or (G) males, in CA3 in (H) females or (I) males. Individual points represent individual animals (N=3-6). Data are presented as the mean ±SEM. One-way ANOVA with Tukey post hoc analysis. Scale bar= 200μm.

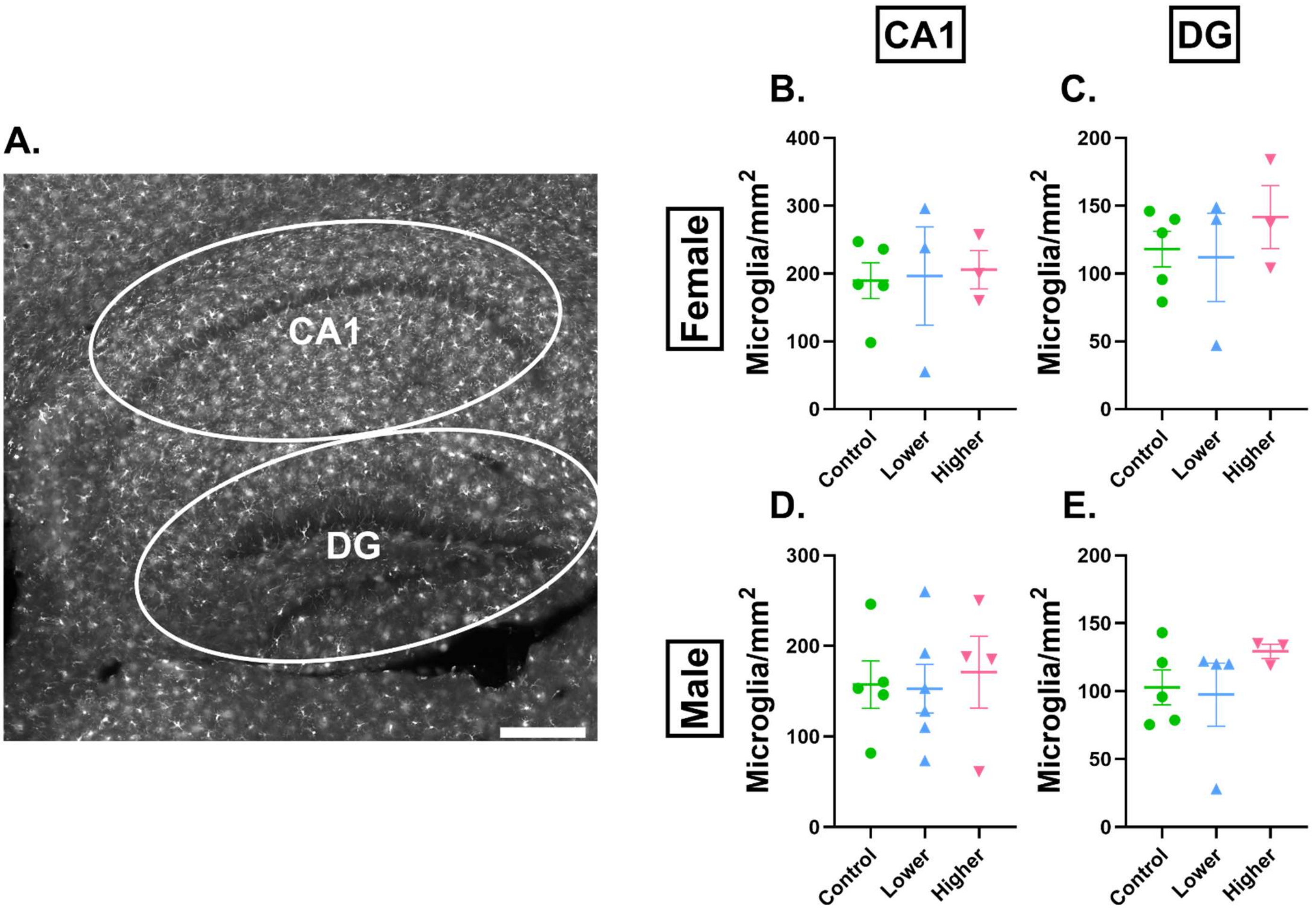

Supplementary Figure S8. PFHxA exposure does not affect microglia density in the hippocampus. (A) Representative image of Iba1 immunolabeling in the CA1 and dentate gyrus. In females, there were no changes in microglia density in the (B) CA1 or the (C) dentate gyrus. In males, there were no changes in microglia density in the (D) CA1 or the (E) dentate gyrus. Individual points represent individual animals (N=3-5). Data are presented as the mean ±SEM. One-way ANOVA with Tukey post hoc analysis.
